# *In-silico* identification and optimization of MMP-9 inhibitors for cerebral ischemia using structure based virtual screening, MD simulation, and binding free energy calculations

**DOI:** 10.1371/journal.pone.0346627

**Published:** 2026-05-14

**Authors:** Jian Zhang, Beibei Zhang, Haisong Feng, Chongxiao Sheng

**Affiliations:** 1 Hubei Provincial Hospital of Traditional Chinese Medicine, Wuhan, Hubei, China; 2 Hubei Key Laboratory of theory and application research of liver and kidney in traditional Chinese Medicine, Affiliated Hospital of Hubei University of Chinese Medicine, Wuhan, Hubei, China; Kwara State University, NIGERIA

## Abstract

Cerebral ischemia is a leading cause of disability and mortality due to the limited therapies of neuroprotection. Matrix metalloproteinase-9 (MMP-9) plays a major role in cerebral ischemia as it breaks down the components of extracellular matrix, which maintains the tissue structure and integrity, making MMP-9 a potential target for therapeutic intervention. The existing inhibitors show poor pharmacokinetics so in this study, we have designed a comprehensive pipeline which combines structure-based pharmacophore modelling, and MD simulation to identify selective MMP-9 inhibitors. We performed virtual screening and then hits were processed by molecular docking, ADMET analysis. Our results identify hit compound CHEMBL3990662 whose stability with MMP-9 was confirmed by MD Simulation. Further the binding free energy was calculated by employing MMGBSA and MMPBSA methods. This study highlights the potential of computational pipeline in the development of MMP-9 inhibitors.

## 1. Introduction

Cerebral ischemia is one of the leading causes of long-term disability and mortality rates worldwide due to the lack of effective therapies which make an urgent need to identify new drug discovery approaches for the development of effective drugs against the disease. The dysregulated metalloproteins, especially MMP-9 plays a key role in pathological role in ischemic injury which shows excessive inflammation, extracellular matrix breakdown and neuronal death. Overexpression of MMP-9 breaks the blood brain barrier, induces neuronal apoptosis, promotes leukocyte infiltration, and amplifies neurodegeneration, which makes it a potential target for drug design [1–4]. Despite extensive efforts to develop inhibitors targeting matrix metalloproteinases, clinical progress has been limited due to the structural similarity among MMP family members, catalytic zinc-dependent binding mechanisms, and challenges associated with achieving favourable pharmacokinetic profiles [5,6].

Recent studies utilized the computational techniques to explore the MMP-9 inhibition mechanism, with many identifying small molecules that are capable of modulating the catalytic zinc-binding environment or blocking substrate access [7–9]. However, the existing studies rely on docking and short simulations which lack deeper understanding of conformational states and ligand induced dynamics. Furthermore, many reported studies rely on limited MD analyses without comprehensive evaluation of conformational sampling, interaction persistence, and binding free energy estimation.

To enhance the robustness of computational prioritization, post-simulation analyses such as principal component analysis (PCA), conformational clustering, and free-energy landscape (FEL) mapping can provide insight into dominant structural states and protein flexibility. In addition, end-point binding free energy methods, including Molecular Mechanics/Generalized Born Surface Area (MM/GBSA) and Molecular Mechanics/Poisson–Boltzmann Surface Area (MM/PBSA), offer quantitative estimates of relative binding affinities and help rank candidate compounds based on energetic stability.

In the present study, we developed a structured computational workflow aimed at prioritizing potential MMP-9–binding compounds and characterizing their structural and dynamic behaviour within the catalytic domain. Receptor-based virtual screening was performed to identify candidate molecules, followed by all-atom molecular dynamics simulations to evaluate complex stability, residue-level interactions, and conformational changes. Binding free energies were further estimated using MM/GBSA and MM/PBSA calculations to support compound ranking and interaction assessment. Additionally, trajectory-based analyses were conducted to examine conformational variability and dominant structural states of the protein–ligand complexes.

This integrated computational framework enables systematic evaluation and prioritization of candidate MMP-9–binding compounds based on structural stability, interaction profiles, and calculated binding energetics. The study is intended to provide mechanistic and computational insights into MMP-9–ligand interactions that may guide future experimental validation studies.

## 2. Methodology

### 2.1 Structure-based pharmacophore generation

The crystal structure of matrix metalloproteinase-9 (MMP-9) bound to a selective inhibitor (PDB ID: 2OVX) was retrieved from the Protein Data Bank. The structure was imported into Maestro (Schrödinger Suite) for preprocessing [10]. Initial refinement was carried out using the Protein Preparation Wizard, which involved assigning proper bond orders, addition of missing hydrogens, optimization of protonation states, and adjustment of the hydrogen-bonding network, followed by restrained minimization to relieve local steric clashes without disturbing the backbone conformation [11,12]. A structure-based pharmacophore hypothesis was developed using the Phase module, which is well suited for deriving pharmacophoric features directly from the three-dimensional arrangement of residues within the active site [13]. Residue selection was not arbitrary; rather, interaction features were derived based on (i) analysis of the crystallographic ligand–protein interactions, (ii) proximity to the catalytic Zn² ⁺ ion, and (iii) documented conserved binding-site residues reported in previous structural studies of MMP-9 inhibitors [14]. Specifically, residues forming the catalytic and substrate-recognition subsites (S1 and S1′), including His226, His401, His405, His411, and Glu402, were considered due to their structural involvement in zinc coordination and ligand stabilization. Interaction features were extracted based on observed hydrogen bonds, hydrophobic contacts, and metal-associated interaction geometry within the co-crystal complex. Pharmacophoric features included hydrogen bond donor/acceptor sites, hydrophobic regions, aromatic rings, and metal-binding features reflecting the zinc-dependent catalytic environment. To assess the discriminative capability of the pharmacophore hypothesis, the model was evaluated using a validation set comprising known MMP-9 inhibitors and a decoy dataset generated to match physicochemical properties while lacking reported activity. Model performance was assessed using receiver operating characteristic (ROC) analysis, ensuring the hypothesis could effectively distinguish active compounds from inactive compounds. The final pharmacophore model was selected based on its ability to recover known actives with high early enrichment while minimizing false positives.

### 2.2 Compound library preparation and pharmacophore-based virtual screening

A virtual screening workflow was applied using the validated pharmacophore model. A dataset comprising 25,083 small molecules was retrieved from ChEMBL. The dataset was constructed by filtering compounds with standard preprocessing steps, including removal of duplicate entries, salts, inorganic species, and compounds violating basic drug-likeness criteria (MW < 150 Da or > 600 Da). The compounds were prepared for virtual screening by Phase database preparation module [13]. Further, Epik was used to generate twenty low energy conformers per compounds to ensure the comprehensive conformational coverage and account for pH-dependent ionization changes, and the energetically unfavourable tautomers were eliminated [12,15]. After database preparation, the pharmacophore hypothesis was utilized for virtual screening using Phase. The screened hits were evaluated based on the phase screen score, which is a combination of vector matching, site matching, steric complementary and RMSD if aligned feature. A Phase Screen Score threshold of ≥ 1.5 was selected to retain high-confidence pharmacophore matches while reducing false positives and maintaining a tractable subset for subsequent docking refinement. This cutoff was chosen based on the distribution of screening scores and to balance sensitivity and selectivity within the dataset.

### 2.3 Molecular docking studies

The crystal structure of the MMP-9 protein (PDB ID: 2OVX) processed in the pharmacophore modelling step was further prepared for docking with the Maestro Protein Preparation Wizard. The preparation protocol contained three steps: preprocessing, optimization, and minimization [11]. For preprocessing, additional hydrogen atoms were added, bond orders were created, alternative conformations evaluated, and non-essential heteroatoms removed from the structure. The protonation and tautomeric states of the ionizable residues were assigned by using PROPKA at the pH 7.0 [16]. The hydrogen bonding networks were then optimised, and a restrained energy minimisation of the MMP-9 crystal structure was conducted under the OPLS force field to eliminate steric clashes [17]. A receptor grid was generated by selecting the co-crystal ligand with centre coordinates set to X = 24.96, Y = 8.68, Z = 51.16 for site-specific docking. The screened hits were prepared using LigPrep, where proper stereochemistry, protonation states, and low-energy conformers were generated at physiological pH [18]. The prepared ligands were docked to MMP-9 receptor using SP mode of glide tool and the results were analyzed based on glide scores.

### 2.4 Drug-likeness evaluation and in silico ADMET prediction

The SwissADME online toolkit was utilized to perform a drug-likeness evaluation of the selected compounds by applying standard drug-likeness rules developed by Lipinski, Veber, Ghose, and Egan, as well as by determining the extent to which the selected compounds meet the guidelines of known small molecule drug categories [19]. The physicochemical characteristics of each compound, which include molecular weight, topological polar surface area (TPSA), lipophilicity (LogP), hydrogen bond donor/acceptor count were evaluated to ensure that they meet the criteria specified by the drug categories for orally active small molecules. Comprehensive evaluation of ADMET (Absorption, Distribution, Metabolism, Excretion, and Toxicity) characteristics was performed to further evaluate the compounds in terms of pharmacokinetics. This evaluation was performed with the use of ADMETLab 3.0 [20]. Evaluation of compounds was conducted based on criteria such as blood-brain barrier penetration, CYP450 interactions, plasma protein binding, risk for hepatotoxicity, risk for mutagenicity, and risk for cardiotoxicity to determine which compounds would have the greatest potential for safety and pharmacokinetic characteristics to be used in the field of neuroprotective therapies for the treatment of cerebral ischemia.

### 2.5 Molecular dynamics simulation

To investigate the dynamic stability and binding persistence of the most promising protein-ligand complex, an all-atom 200 ns molecular dynamics (MD) simulation was performed using NAMD 3.0 [21]. The initial coordinates for the simulation were obtained from the highest-ranking docking pose. System preparation and parameterization were conducted using the AmberTools24 suite. The protein was modelled using the AMBER ff14SB force field, which provides refined parameters for amino acid side chains and backbone dynamics [22]. For the small-molecule ligand, topology and parameter files were generated through the antechamber module using the General Amber Force Field (GAFF) [23]. Atomic partial charges for the ligand were assigned using the AM1-BCC semi-empirical method to ensure an accurate representation of the electronic environment [24]. The protein-ligand complex was centred in a cubic box and solvated with TIP3P water molecules, maintaining a minimum buffer distance of 10 Å from the protein surface to the box boundaries [25]. To simulate physiological conditions, the system was neutralized, and an ionic strength of 0.15 M NaCl was established using Joung-Cheatham ion parameters [26]. Energy minimization was performed for 10,000 steps using the conjugate gradient algorithm to resolve steric clashes. Subsequently, the system gradually heated to 310 K over 100 ps in the NVT ensemble. Equilibration was then carried out in the NPT ensemble (1 atm, 310 K) for 1 ns to stabilize the system density. The production MD run was executed for 100 ns with a time step of 2 fs. Long-range electrostatic interactions were treated using the Particle Mesh Ewald (PME) method, while short-range van der Waals interactions were calculated with a 12 Å cutoff [27]. The SHAKE algorithm was employed to constrain all covalent bonds involving hydrogen atoms [28]. Temperature and pressure were maintained using a Langevin thermostat and a Langevin piston, respectively. Ultimately, the trajectory was analyzed by using the BIO3D library of R [29].

### 2.6 Binding free energy calculations

The binding free energies of the protein-ligand complexes were calculated using the MMPBSA.py module provided in AmberTools. A total of 50 snapshots were extracted from the last 30 ns of the MD trajectory for analysis. The binding free energy was estimated using the Single Trajectory Protocol, where the coordinates for the receptor and ligand were extracted from the trajectory of the complex. The energy is defined by the following equations:


$${\Delta \text{G}}_{bind}=\;{\Delta \text{G}}_{complex}-\;{\Delta \text{G}}_{receptor}-\;{\Delta \text{G}}_{ligand}$$

For each individual component, the free energy was calculated as:


$$\Delta \text{G}=\;{\Delta \text{E}}_{bond}+{\Delta \text{E}}_{vdw}+{\Delta \text{E}}_{ele}+{\Delta \text{G}}_{pol}+\;{\Delta \text{G}}_{np}$$

ΔE_bond_, ΔE_vdw_, ΔE_ele_ represent the internal (bond, angle, dihedral), van der Waals, and electrostatic interactions from the FF19SB. ΔG_pol_ is the polar solvation contribution, calculated using the Poisson-Boltzmann (PB) and Generalized Born (GB) models. ΔG_np_ is the non-polar solvation energy, determined from the solvent-accessible surface area (SASA) using the LCPO algorithm.

#### 2.6.1 Per-residue energy decomposition.

To provide a deeper insight into the molecular basis of binding, energy decomposition was performed using the MM-GBSA method. This allows for the breakdown of the total binding energy into contributions from individual residues by considering the interactions between each protein residue and the ligand. The total energy contribution of a residue is the sum of its internal, van der Waals, electrostatic, and solvation energy components:


$${\Delta \text{G}}_{residue}=\;{\Delta \text{E}}_{vdw}+{\Delta \text{E}}_{ele}+{\Delta \text{G}}_{pol}+\;{\Delta \text{G}}_{np}$$

## 3. Results and discussion

### 3.1 Pharmacophore hypothesis development

**Fig 1:**
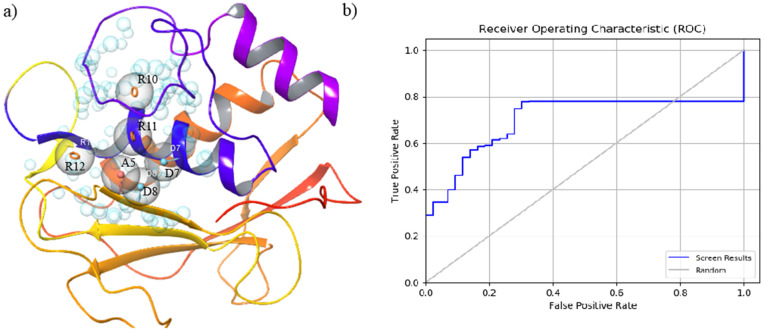
(a) Structure-based pharmacophore model derived from the MMP-9 active site, showing aromatic (orange), hydrogen bond donor (blue), and hydrogen bond acceptor (red) features mapped within the catalytic pocket. (b) Receiver operating characteristic (ROC) curve evaluating pharmacophore performance against active and decoy compounds (AUC = 0.70).

The crystal structure of MMP-9 (PDB ID: 2OVX) was prepared for pharmacophore hypothesis generation using the Phase module. Binding site features were derived based on the crystallographic ligand–protein interaction pattern and spatial arrangement of residues within the catalytic pocket. The final pharmacophore model consisted of seven features, including three aromatic ring features, three hydrogen bond donor features, and one hydrogen bond acceptor feature (Fig 1a). These features correspond to residues positioned within the catalytic and substrate-recognition regions of MMP-9. Specifically, interaction points were mapped in proximity to residues corresponding to aromatic regions (R10, R11, R12), hydrogen bond donor regions (D7, D8), and hydrogen bond acceptor regions (A5), reflecting the interaction geometry observed in the co-crystal structure. The spatial arrangement of these features is summarized in Table 1. To evaluate the discriminative ability of the pharmacophore model, validation was performed using a dataset comprising known MMP-9 inhibitors and decoy compounds. Receiver operating characteristic (ROC) analysis yielded an area under the curve (AUC) value of 0.70 (Fig 1b), indicating moderate classification performance above random selection.

**Table 1:** The phase scores and coordinates of the pharmacophore hypothesis.

Rank	Features	Score	X	Y	Z
1	R10	−1.41	24.3082	2.257	52.7882
2	R11	−1.38	26.0343	6.6231	51.1221
3	D8	−0.66	28.6364	10.5538	48.5609
4	A5	−0.54	27.7268	10.54	50.7784
5	R12	−0.2	22.4903	14.0517	54.1211
6	D7	−0.19	25.4934	8.9978	46.5939

### 3.2 Virtual screening

The developed pharmacophore model was employed to screen the ChEMBL database. A compound was considered a hit if it matched at least four features of the pharmacophore [30]. The screened compounds were then ranked using the Phase screen score, a composite measure combining the volume score, RMSD site matching, and vector alignment. A high vector score (ranging from −1.0 to 1.0) indicated better alignment between the pharmacophore and the ligand. The volume score, which ranged from 0.0 to 1.0, quantified the overlap between the reference ligand and the aligned ligands. Higher volume scores (close to 1.0) indicate greater overlap, and the phase screen score threshold for hit identification was established to 1.5. Therefore, 278 compounds from the ChEMBL database met the hit selection criteria. Thus, these were identified as potentially good hits for further analysis.

### 3.3 Molecular docking studies

**Fig 2:**
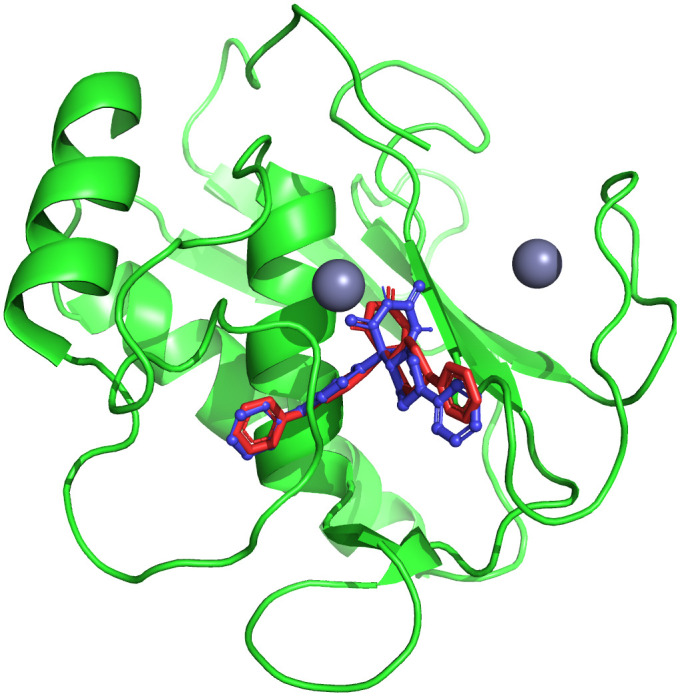
Superimposition of the native co-crystal ligand (red) and the redocked pose (blue) within the active site of MMP-9 (RMSD = 0.217 Å).

**Fig 3:**
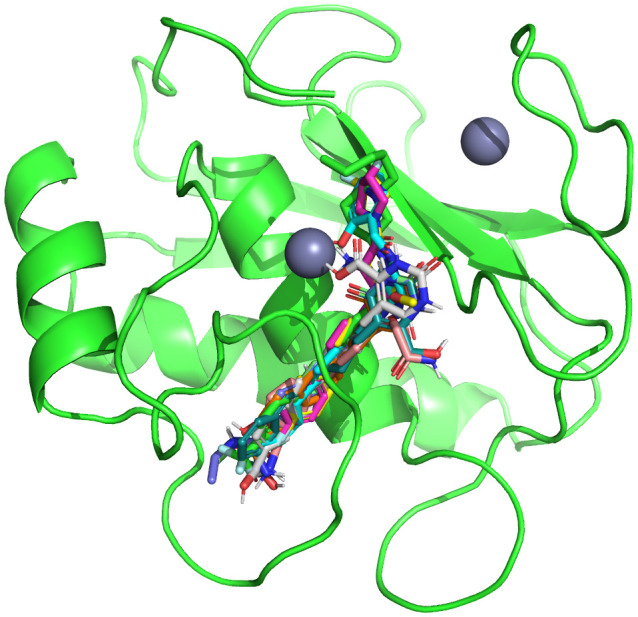
Superimposition of the docked compounds on the co-crystal ligand with RMSD < 0.3 Å.

To study the binding mechanisms of the selected hits, molecular docking studies were carried out. Prior to the docking of hit compounds with the MMP-9 receptor, the redocking of co-crystal ligand was performed to validate the docking protocol. The redocked pose showed a RMSD of 0.217 Å with the native pose as shown in Fig 2. The docking was performed using the refined MMP-9 structure, and the resulting binding poses were analyzed for interaction patterns within the active site [31]. The 10 most promising compounds, based on their Glide scores, had high affinity for MMP-9 indicated by the low value of their Glide scores, supporting the strength of their interaction with MMP-9 when assessed using Discovery Studio [32]. MMP-9 is a zinc-dependent metalloprotease, and the catalytic Zn² ⁺ ion located within the active site was retained during docking calculations. The zinc ion is coordinated by conserved histidine residues and plays a central role in ligand stabilization within the catalytic cleft. Therefore, ligand interactions were evaluated not only for hydrogen bonding and hydrophobic contacts but also for potential interactions in proximity to the catalytic zinc environment. The docking analysis provided extensive data regarding the interaction of the ten compounds with MMP-9 including hydrogen bond and hydrophobic interactions with amino acids, including Arg424, Thr426, Gly186, His401, Leu188, and Tyr423, which all are critical amino acids for effective MMP-9 inhibition. Additional hydrophobic interactions with amino acids including Leu418 and Phe110 also supported the formation of stable protein-ligand complexes. When aligned in the binding pocket of MMP-9, all ten of the compounds exhibited consistent patterns of binding interaction. The superimposition of the compounds is shown in Fig 3. The interaction analysis of the MMP-9 binding affinities of the scaffolds indicated that many of their interactions are hydrogen bonding and hydrophobic in nature. Table 2 contains an overview of Glide scoring and key interactions (hydrogen bonding and hydrophobic) with MMP-9 for each of the scaffolds. Interestingly, CHEMBL1184827 and CHEMBL3990662 had the highest binding affinity for MMP-9, as they formed hydrogen bonds with the important amino acids (Arg424, Thr426 and Tyr423) and had hydrophobic interactions that helped stabilise the complexes.

**Table 2:** Molecular docking results of top 10 compounds.

Ids	Structure	Glide score	Hydrogen Bonds (Distance (Å))	Zn Interaction	Hydrophobic Interaction
CHEMBL3990662		−9.96	Arg424 (1.98), Thr426 (2.56), Tyr423 (2.78), Gly186 (2.12)	Pi Cation	Leu418, His401, Leu188
CHEMBL1184827		−9.93	His405 (2.72), Leu188 (2.60)	Pi Cation	Arg424, Val398, His401, Ala191, Phe110
CHEMBL1194647		−9.73	Gly186 (2.27), Val398 (3.32), Arg424 (2.10), Ala417 (2.22), Tyr423 (2.77)	Pi Cation	Leu188, His401, Gln402, Leu418
CHEMBL3990207		−9.60	Ala417 (2.74), Tyr423 (2.84), Gly186 (2.02), Glu416 (2.90), Pro423 (2.77)	Pi Cation	Leu418, His401, Leu188
CHEMBL3991298		−9.59	Pro430 (2.88), Glu416 (2.74), Tyr423 (2.85), Gly186 (2.01)	Pi Cation	Leu418, His401, Leu188
CHEMBL3990161		−9.35	Thr426 (1.75), His401 (3.02), His411 (2.21), Gly186 (2.51)	Pi Cation	Arg424, Leu418, Leu397, Leu188
CHEMBL3991299		−9.34	Gly186 (2.02), Tyr423 (2.88), Arg424 (1.98), Thr426 (3.11)	Pi Cation	Leu188, Leu397, Leu418
CHEMBL546070		−9.32	Ala189 (1.80), Leu188 (2.72), Tyr420 (2.63), Thr426 (3.23), Arg424 (2.72)	Pi Cation	His405, His411, Phe110, His401, Leu418, Glu416
CHEMBL1181759		−9.58	His405 (2.77), Leu188 (2.80)	Pi Cation	Leu397, Val398, His401, Leu418, Arg424,
CHEMBL547206		−9.28	His405 (1.98), His411 (3.06), Ala189 (1.91), His401 (2.06)	Pi Cation	Leu187, Leu188, Leu418, Arg424, Leu397, Val398, Tyr423

### 3.4 Drug-likeness prediction

Several Drug-Likeness Profiles for the Top 10 Compounds, were evaluated based on Lipinski’s “Rule of Five”, PAINS alert, and Brenk’s evaluation of toxicological properties [33]. The rule of five is often used in the drug discovery to estimate the likelihood of oral bioavailability. Lipinski’s rule of five states that, if the molecular weight (MW) is greater than 500 Daltons, the compound will be less likely to have good oral bioavailability. Therefore, all Compounds met the criteria for inclusion in a class of compounds likely to return favourable absorption profiles. In terms of both hydrogen bond donors and acceptors, there were varying values, but all values were within the accepted limits for bioactivity. For Example, CHEMBL3990662 had five hydrogen bond acceptors and three hydrogen bond donors, and CHEMBL1184827 had Six hydrogen bond acceptors and one hydrogen bond donor and still maintained a suitable design for drug-like characteristics. The topological polar surface area (TPSA) values of the screened compounds ranged from 53.7 to 146 Å² (Table 3). TPSA is a descriptor of molecular polarity and is frequently used as an indirect indicator of passive membrane permeability and, to some extent, aqueous solubility; however, it does not directly measure solubility. In general, compounds with TPSA values below ~140 Å² are more likely to exhibit favourable passive permeability, whereas values above this threshold may be associated with reduced membrane and blood–brain barrier penetration. Accordingly, compounds at the upper end of the observed range (e.g., CHEMBL3990662; TPSA = 146 Å²) may face permeability constraints despite potentially improved polarity-related properties. These findings were therefore interpreted alongside other physicochemical parameters (LogP and predicted LogS) and ADMET predictions rather than TPSA alone. LogP was also calculated, ranging from 0.82–3.90, and indicates that the compounds exhibit a balance between hydrophobic (0.82) and hydrophilic (3.90) characteristics. This balance is necessary for membrane permeability. The high LogP (3.90) of CHEMBL546070 indicates a relatively high lipophilicity but still within a tolerable range for bioavailability. The calculated solubility (LogS) values also range from −2.8 to −5.6, indicating these compounds are potential drug candidates. Alerts for the PAINS category were found in two compounds, CHEMBL1194647 and CHEMBL547206, both of which had one alert, indicating that further validation of specificity may be necessary despite their apparent potent bioactivities. All compounds passed the evaluation of Brenk’s rules, which evaluate the safety of compounds by looking at toxicological alerts, therefore indicating that they are safe candidates for continued improvement. Table 3 summarize the drug-like properties (molecular weights, numbers of hydrogen bond donors and acceptors, TPSA, LogP, etc.) for each of the top candidate compounds.

**Table 3:** The physicochemical properties and drug-likeness criteria of the selected compounds.

Ids	MW	HBA	HBD	TPSA	LogP	LogS	Lipinski	PAINS	Brenk	SA
CHEMBL3990662	352	5	3	146	0.82	−2.8	Yes	0	0	2.72
CHEMBL1184827	388	6	1	72.6	2.41	−3.7	Yes	0	0	3.81
CHEMBL1194647	370	4	3	117	1.46	−3.6	Yes	1	0	2.65
CHEMBL3990207	380	5	3	134	1.35	−3.3	Yes	0	0	2.86
CHEMBL3991298	366	5	3	132	1.15	−3.1	Yes	0	0	2.77
CHEMBL3990161	380	6	2	140	2.70	−3.9	Yes	0	0	2.90
CHEMBL3991299	380	6	2	140	2.67	−3.9	Yes	0	0	2.90
CHEMBL546070	498	7	3	88	3.90	−5.6	Yes	0	0	4.16
CHEMBL1181759	374	4	2	91	2.06	−3.1	Yes	0	0	3.69
CHEMBL547206	373	2	3	53.7	3.36	−4.8	Yes	1	0	3.14

### 3.5 In silico ADMET prediction

ADMET predictions of selected compounds were conducted using ADMETLab3.0 which provided an in-silico investigation of absorption, distribution, metabolism, excretion and toxicity (ADMET) profiles [20]. All the compounds had favourable potential for absorption (HIA positive) into the bloodstream and thus are likely to be able to access their target tissues. The distribution of compounds (volume of distribution (VD)), ranged from 0.155 L kg ⁻ ¹ to 2.395 L kg ⁻ ¹. Compounds CHEMBL1194647 (1.969) and CHEMBL547206 (2.395), showed the highest VD values and therefore have a higher likelihood of distributing widely throughout all tissues, including tumour sites. VD values are advantageous for therapeutic effectiveness, but they also raise the potential risks associated with accumulation of the drug into non-target tissues and increased likelihood of side effects. Compounds such as CHEMBL3990161 with a lower VD value may pose a lesser risk for non-specific exposure. All the compounds demonstrated predictably to be able to cross the blood-brain-barrier (BBB); therefore, they have a definite advantage for treating disorders of the Central Nervous System (CNS) or brain cancer, as this route offers high concentrations delivered directly to brain tissue while having potentially less impact on systemic toxicity. However, CNS penetration may produce CNS adverse effects, such as neurotoxicity and sedation, which require monitoring in future studies. Plasma protein binding (PPB) is variable among the compounds studied, but there are lower PPB in some compounds but higher PPB in others. For example, CHEMBL3990662 (PPB = 0.454) and CHEMBL3991299 (PPB = 0.361) have lower plasma protein binding than other compounds, indicating freer drug is available for pharmacological activity. Conversely, some compounds, such as CHEMBL546070 (PPB = 4.591), have higher PPB, leading to lower concentrations of the free active species in circulation, which may reduce toxicity but also subsequently decrease pharmacological action. Similarly, with respect to metabolism, the compounds studied displayed substantial variations in their ability to interact with cytochrome P450 family enzymes (CYPs). As indicated above, several compounds, including CHEMBL1184827, have been shown to interact with CYP3A4, CYP2D6 and CYP2C19, which are involved in the metabolism of a variety of drug classes. It is worth noting that about 50% of all drugs are metabolized by CYP3A4, therefore it is important to consider these interactions when predicting drug-drug interactions for compounds of interest. Because compounds can interact with CYP enzymes, it is important to note these interactions may have an impact on the efficacy and toxicity of drugs that will be used due to altered plasma levels. In silico mutagenicity predictions based on the Ames toxicity model indicated that several compounds were classified as predicted non-mutagenic, including CHEMBL1194647, CHEMBL3990161, and CHEMBL3991299. In contrast, CHEMBL1181759 and CHEMBL547206 were predicted to have potential mutagenicity risk according to the ADMETlab classification. These results represent computational estimates and should be interpreted cautiously, as experimental toxicological validation would be required to confirm safety profiles. The evaluation of hepatotoxicity for the selected compounds indicated that there were no significant hepatotoxic effects associated with any of the compounds, indicating these compounds may be safer to use clinically. ADMET predictions for the compounds are given in Table 4 and include details on the pharmacokinetic parameters and toxicological data for each of the compounds.

**Table 4:** The ADMET profiles of the selected compounds predicted by ADMETLab 3.0.

	Absorption	Distribution			Metabolism							Excretion	Toxicity
	HIA	VD	BBB	PPB	Substrate		Inhibitor					Total Clearance	Ames toxicity
		(L kg-1)			2D6	3A4	1A2	2C19	2C9	2D6	3A4	(mL min-1nkg-1)	
CHEMBL3990662	Yes	0.207	Yes	Yes	---	---	---	---	---	---	---	2.311	No
CHEMBL1184827	Yes	1.357	Yes	Yes	+	+++	--	---	---	---	–	5.03	Yellow
CHEMBL1194647	Yes	1.969	Yes	Yes	---	---	---	---	---	---	---	7.189	No
CHEMBL3990207	Yes	0.229	Yes	No	---	---	---	---	---	---	---	3.262	Yellow
CHEMBL3991298	Yes	0.217	Yes	No	---	---	---	---	---	---	---	2.966	Yellow
CHEMBL3990161	Yes	0.155	Yes	No	---	---	---	---	---	---	---	0.454	No
CHEMBL3991299	Yes	0.168	Yes	No	---	---	---	---	---	---	---	0.361	No
CHEMBL546070	Yes	1.119	Yes	No	---	++	---	---	--	+++	+++	4.591	No
CHEMBL1181759	Yes	0.975	Yes	Yes	---	–	---	---	---	---	--	4.429	Yes
CHEMBL547206	Yes	2.395	Yes	No	–	---	---	---	---	---	---	6.552	Yes

+++ strong predicted interaction, ++ moderate interaction, + weak interaction, – very weak interaction, -- low probability, --- predicted non-interaction. HIA: Human Intestinal Absorption; VD: Volume of Distribution; BBB: Blood–Brain Barrier permeability; PPB: Plasma Protein Binding; Ames toxicity prediction categories: “Yes” = predicted mutagenic, “No” = predicted non-mutagenic, “Yellow” = moderate/uncertain mutagenicity risk.

### 3.6 Binding mode analysis

**Fig 4:**
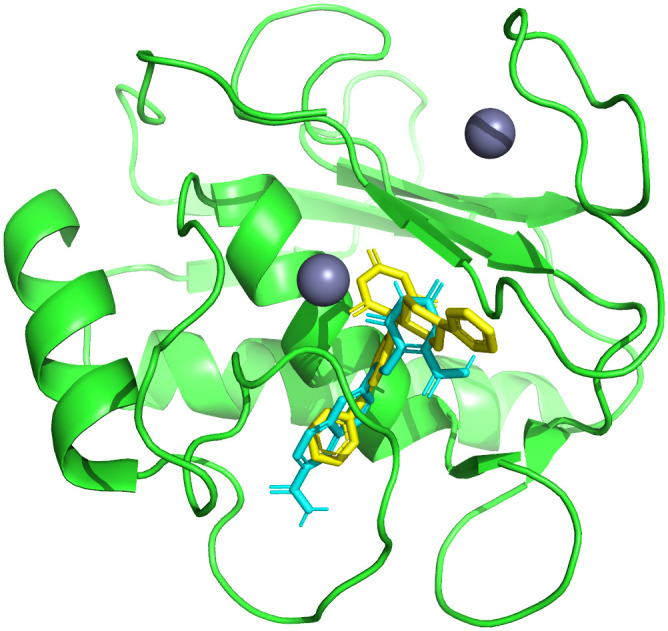
Superimposed binding modes of the co-crystal ligand (yellow) and CHEMBL3990662 (cyan) within the catalytic domain of MMP-9.

Among the screened compounds, CHEMBL3990662 exhibited the most favourable Glide docking score (−9.96 kcal/mol), indicating the strongest predicted binding affinity toward the catalytic domain of MMP-9 among the evaluated candidates. Detailed interaction analysis revealed that CHEMBL3990662 forms multiple stabilizing hydrogen bonds with key active-site residues, including Arg424 (1.98 Å), Thr426 (2.56 Å), Tyr423 (2.78 Å), and Gly186 (2.12 Å), which are located within the substrate-binding cleft. In addition, π–cation interactions were observed within the catalytic pocket, along with hydrophobic contacts involving Leu418, His401, and Leu188, suggesting favourable accommodation within the active-site architecture. Importantly, the binding orientation of CHEMBL3990662 overlapped substantially with that of the co-crystal ligand within the catalytic cavity (Fig 4), indicating a comparable positioning relative to the zinc-coordinating region and surrounding substrate-recognition residues. The similar binding mode, combined with its superior docking score and stable interaction network, supported the selection of CHEMBL3990662 for subsequent molecular dynamics simulations. The co-crystal ligand was included as a reference system to provide a structural baseline for comparative evaluation of stability, conformational behaviour, and binding free energy calculations.

### 3.7 MD simulation

**Fig 5:**
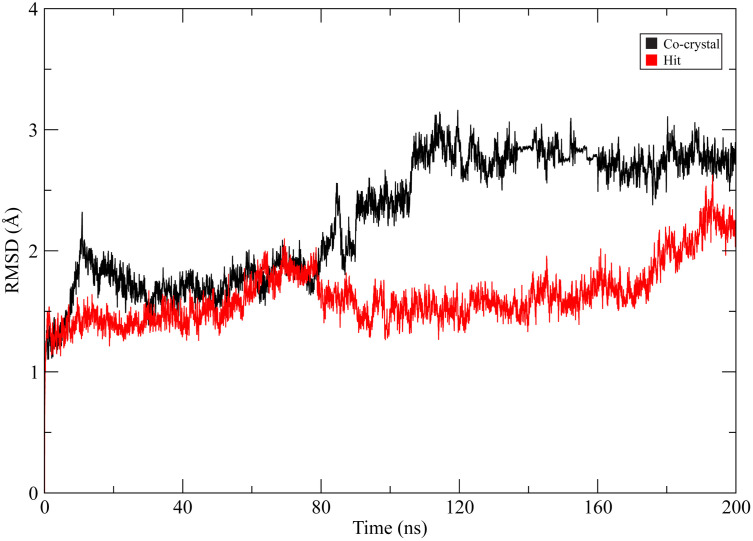
Backbone RMSD profiles of MMP-9 during 200 ns molecular dynamics simulations. The co-crystal ligand complex is shown in black, and the CHEMBL3990662 complex (hit compound) is shown in red.

To evaluate the structural stability of the protein–ligand complexes, 200 ns molecular dynamics simulations were performed for both the CHEMBL3990662–MMP-9 complex and the co-crystal ligand–MMP-9 complex. The backbone RMSD of the protein was calculated throughout the simulation [34]. As shown in Fig 5 both systems exhibited initial equilibration within the first 20–30 ns. The co-crystal complex displayed a gradual increase in RMSD, reaching values between approximately 2.6 and 2.9 Å after ~90 ns, where it remained relatively stable with moderate fluctuations for the remainder of the trajectory. In contrast, the CHEMBL3990662 complex maintained comparatively lower RMSD values throughout most of the simulation, fluctuating primarily between ~1.4 and 1.8 Å up to approximately 160 ns. A modest increase was observed during the final portion of the trajectory (~170–200 ns), with RMSD values approaching ~2.1–2.3 Å; however, no abrupt structural deviations were detected. Overall, the reduced backbone RMSD observed for the CHEMBL3990662 complex relative to the co-crystal system suggests stable accommodation of the ligand within the catalytic domain and absence of major conformational disruption. These findings support the structural stability of the selected hit compound during extended simulation.

**Fig 6:**
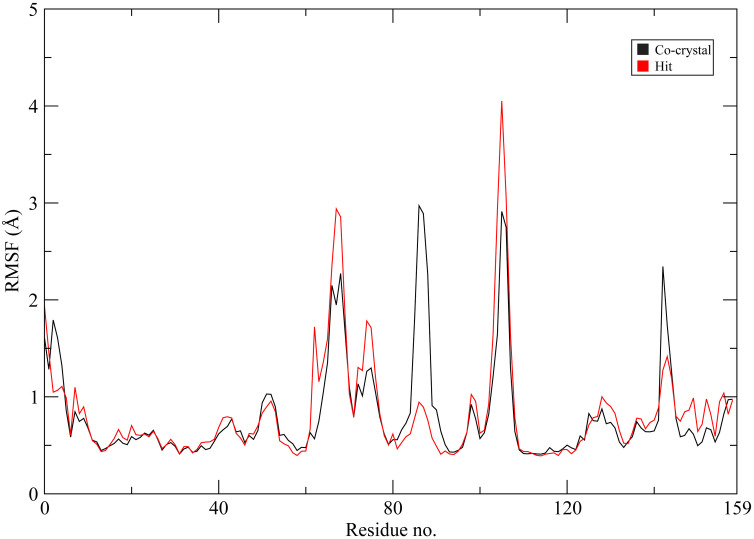
Residue-wise RMSF profiles of MMP-9 during 200 ns molecular dynamics simulations. The co-crystal ligand complex is shown in black, and the CHEMBL3990662 complex (hit compound) is shown in red.

To further investigate local structural flexibility, residue-wise root mean square fluctuation (RMSF) values were calculated for both complexes [34]. As shown in Fig 6, most residues in both systems exhibited relatively low fluctuations, generally ranging between ~0.4 and 1.0 Å, indicating overall structural stability of the protein backbone. Regions corresponding to loop segments showed comparatively higher flexibility, as expected for solvent-exposed areas. Notable fluctuations were observed around residues ~70–75 and ~100–105 in the CHEMBL3990662 complex, where RMSF values reached approximately 3.0 Å and 4.0 Å, respectively. In contrast, the co-crystal complex displayed pronounced flexibility around residue ~85 (~3.0 Å) and around ~135–140 (~2.3 Å). Importantly, residues located within the catalytic pocket and substrate-binding cleft maintained relatively moderate fluctuations in both systems, suggesting preservation of the active-site architecture throughout the simulation. Overall, the RMSF profiles indicate that ligand binding did not induce excessive destabilization of the protein structure. Differences in flexibility were primarily confined to loop regions rather than core catalytic residues.

**Fig 7:**
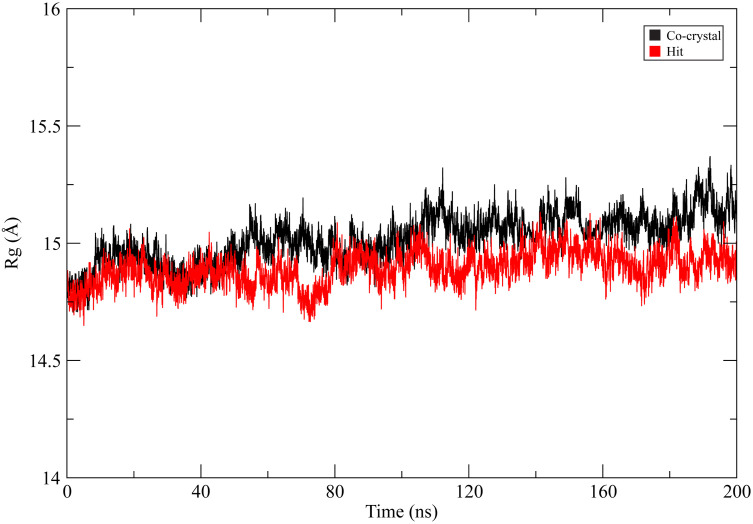
Radius of gyration (Rg) profiles of MMP-9 during 200 ns molecular dynamics simulations. The co-crystal ligand complex is shown in black, and the CHEMBL3990662 complex (hit compound) is shown in red.

To evaluate the overall compactness of the protein structure during simulation, the radius of gyration (Rg) was calculated for both complexes [35]. As shown in Fig 7, both complexes maintained relatively stable Rg values throughout the simulation, fluctuating within a narrow range of approximately 14.7–15.2 Å. The co-crystal complex exhibited slightly higher average Rg values (~15.0–15.2 Å), whereas the CHEMBL3990662 complex showed marginally lower and more consistent values (~14.8–15.0 Å). No significant structural expansion or compaction events were observed in either system during the simulation period. The stable Rg profiles indicate preservation of the global fold of MMP-9 in both complexes. The comparable compactness further supports the structural stability observed in RMSD and RMSF analyses, suggesting that binding of CHEMBL3990662 does not induce large-scale conformational destabilization of the protein.

**Fig 8:**
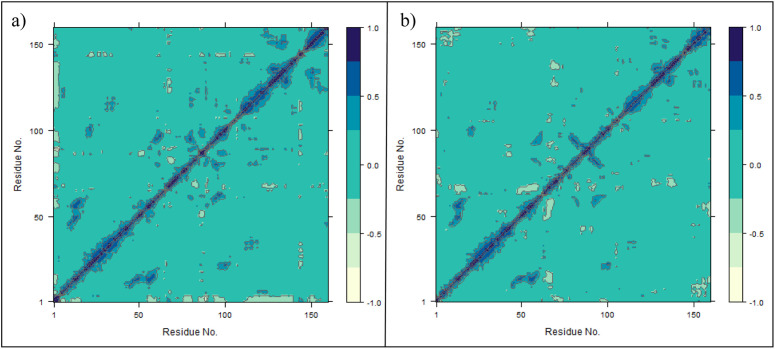
Dynamic cross-correlation matrices (DCCM) of MMP-9 during 200 ns molecular dynamics simulations. **(a)** Co-crystal ligand complex and **(b)** CHEMBL3990662 complex. Positive correlations are shown in blue, negative correlations in light shades, and near-zero correlations in green.

To investigate residue–residue correlated motions during the simulation, dynamic cross-correlation matrix (DCCM) analysis was performed for both complexes [36]. The DCCM describes the extent to which atomic fluctuations of residue pairs are correlated (positive values), anti-correlated (negative values), or uncorrelated during the trajectory. As shown in Fig 8a (co-crystal complex) and Fig 8b (CHEMBL3990662 complex), both systems exhibit strong positive correlations along the main diagonal, corresponding to local backbone connectivity. Outside the diagonal region, most residue pairs display near-neutral correlations, indicating absence of large-scale concerted motions. Localized regions of moderate positive and negative correlations were observed in both systems, particularly within loop segments and flexible regions. The overall correlation patterns remain broadly similar between the two complexes, suggesting that binding of CHEMBL3990662 does not induce major alterations in global dynamic communication within the protein structure. Minor differences in specific off-diagonal regions indicate subtle variations in correlated motions; however, no pronounced long-range anti-correlated domains were observed in either system. These results are consistent with the RMSD and RMSF analyses, supporting preservation of the overall dynamic behaviour of MMP-9 upon binding of the hit compound.

**Fig 9:**
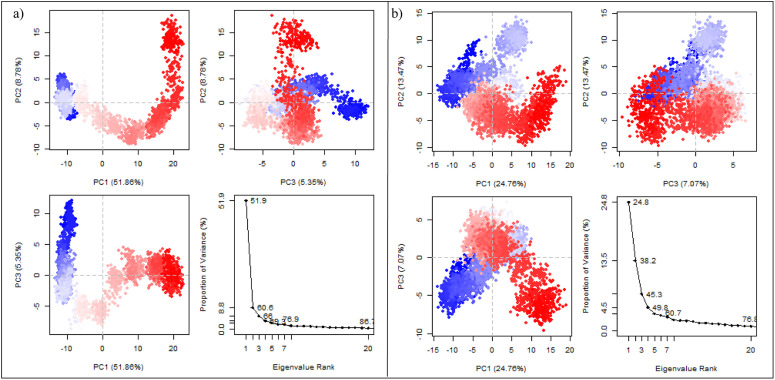
Principal component analysis (PCA) of MMP-9 dynamics. **(a)** Co-crystal ligand complex and **(b)** CHEMBL3990662 complex. Upper panels show 2D projections of PC1 vs PC2 and PC3; lower panels display eigenvalue distributions and cumulative variance contribution.

To explore large-scale collective motions during the simulations, principal component analysis (PCA) was performed on the Cα atom trajectories of both complexes. The projections of the first three principal components (PC1–PC3) and the corresponding eigenvalue distributions are shown in Fig 9. For the co-crystal complex (Fig 9a), PC1 accounted for 51.88% of the total variance, while PC2 and PC3 contributed 8.78% and 5.35%, respectively. Thus, the first three principal components captured approximately 66% of the total motion, indicating that the system’s conformational dynamics were largely dominated by a single principal motion along PC1. In contrast, the CHEMBL3990662 complex (Fig 9b) exhibited a more distributed variance profile, with PC1 accounting for 24.76%, PC2 for 13.47%, and PC3 for 7.07% of the total variance. The first three components collectively captured approximately 45% of the motion, suggesting a broader distribution of conformational sampling across multiple collective modes. The two-dimensional projections reveal that both systems sample distinct conformational clusters over the course of the simulation. The co-crystal complex shows a more elongated distribution primarily along PC1, whereas the CHEMBL3990662 complex displays a comparatively dispersed conformational landscape. These observations indicate differences in the dominant collective motions between the two complexes, while overall structural stability is maintained, as supported by RMSD and Rg analyses.

**Fig 10:**
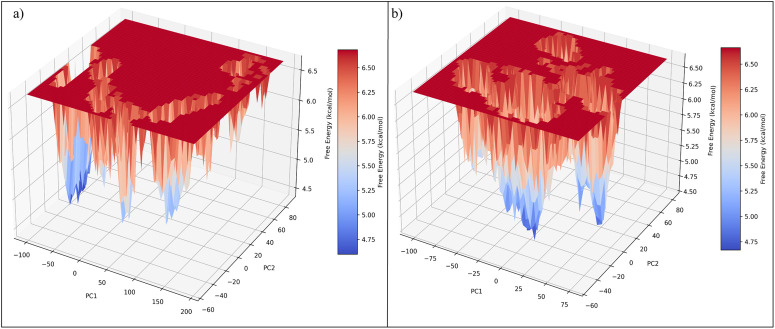
Free energy landscapes (FEL) of MMP-9 complexes constructed using PC1 and PC2 as reaction coordinates. **(a)** Co-crystal ligand complex and **(b)** CHEMBL3990662 complex. Colour scale represents free energy (kcal/mol), where blue regions indicate low-energy, highly populated conformational states.

To further characterize the conformational space sampled during the simulations, free energy landscapes (FEL) were constructed using the first two principal components (PC1 and PC2) as reaction coordinates. As shown in Fig 10a (co-crystal complex) and Fig 10b (CHEMBL3990662 complex), both systems exhibit multiple energy minima distributed across the conformational space. The calculated free energy values range approximately between 4.5 and 6.7 kcal/mol for both complexes. Distinct low-energy basins (blue regions) correspond to the most populated conformational states sampled during the trajectory. The co-crystal complex displays relatively well-defined energy minima localized within specific regions of the PC1–PC2 space, indicating dominant conformational states. In comparison, the CHEMBL3990662 complex shows multiple accessible low-energy basins distributed across the landscape, suggesting sampling of several energetically favourable conformations during the simulation. Importantly, no large-scale destabilization or high-energy transitions were observed in either system. The presence of stable low-energy basins in both complexes supports the structural stability inferred from RMSD, RMSF, and Rg analyses. Overall, the FEL results indicate that the CHEMBL3990662 complex maintains energetically favourable conformational states comparable to the reference system.

### 3.8 Binding free energy calculations

To quantitatively estimate binding affinities and further support compound prioritization, end-point binding free energy calculations were performed using MM/GBSA and MM/PBSA methods (Table 5). In the gas-phase energy components, both complexes exhibited favourable van der Waals (ΔEvdw) and electrostatic (ΔEele) contributions. The CHEMBL3990662 complex showed slightly stronger electrostatic interactions (−15.54 kcal/mol) compared to the co-crystal system (−9.83 kcal/mol), while van der Waals contributions were comparable between the two systems (−52.56 vs −52.10 kcal/mol). As a result, the total gas-phase interaction energy (ΔGgas) was more favourable for the CHEMBL3990662 complex (−68.11 kcal/mol) relative to the co-crystal complex (−61.93 kcal/mol). Upon inclusion of solvation effects, both GB and PB models indicated favourable overall binding free energies. The MM/GBSA total binding free energy (ΔGGB) was calculated as −45.17 kcal/mol for CHEMBL3990662 and −43.78 kcal/mol for the co-crystal ligand. Similarly, MM/PBSA calculations yielded ΔGPB values of −12.08 kcal/mol for CHEMBL3990662 and −6.21 kcal/mol for the co-crystal system. Although the differences between the two systems are moderate, the consistently more negative binding free energies observed for CHEMBL3990662 in both GB and PB models suggest favourable interaction energetics within the MMP-9 binding pocket. These results support the docking and molecular dynamics findings, indicating stable and energetically favourable binding of the selected hit compound.

**Table 5:** MM/GBSA and MM/PBSA binding free energy components (kcal/mol) for the co-crystal ligand and CHEMBL3990662 complexes with MMP-9.

	Co-crystal	CHEMBL3990662
ΔE_vdw_	−52.10	−52.56
ΔE_ele_	−9.83	−15.54
ΔE_GB_	23.94	28.71
ΔE_surf_	−5.79	−5.77
ΔE_PB_	32.18	33.66
ΔE_NP_	−30.67	−30.93
ΔE_Dis_	54.21	53.29
ΔG_gas_	−61.93	−68.11
ΔG_solGB_	18.15	22.93
ΔG_solPB_	55.72	56.02
ΔG_GB_	−43.78	−45.17
ΔG_PB_	−6.21	−12.08

**Fig 11:**
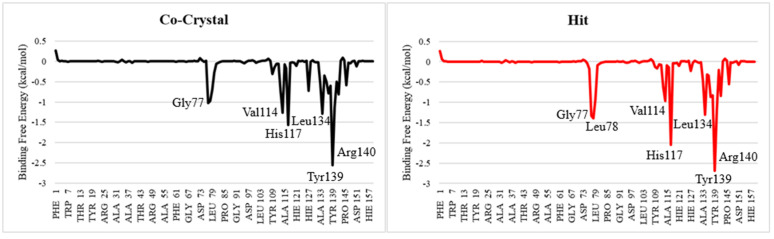
Per-residue binding free energy decomposition (MM/GBSA) for co-crystal ligand and CHEMBL3990662 complexes with MMP-9. Negative values indicate favourable energetic contributions to binding.

To identify key residues contributing to ligand binding, per-residue energy decomposition was performed using the MM/GBSA framework. As shown in Fig 11, several residues within the binding pocket contribute favourably to ligand stabilization in both complexes. In the co-crystal system, notable negative energy contributions were observed for Gly77, His117, Val114, Leu134, Tyr139, and Arg140. These residues are located within or proximal to the catalytic cleft and substrate-recognition region. Similarly, in the CHEMBL3990662 complex, the same residues exhibited favourable energetic contributions. In particular, His117, Tyr139, and Arg140 showed comparatively stronger negative binding energy contributions relative to the co-crystal system, indicating enhanced local interaction stabilization in these regions. Contributions from hydrophobic residues such as Val114 and Leu134 further support the importance of nonpolar contacts in maintaining ligand accommodation within the binding pocket. Overall, the per-residue decomposition results indicate that CHEMBL3990662 engages key catalytic and surrounding residues in a manner comparable to the reference ligand, with modest differences in interaction strength for specific residues. These findings are consistent with the total binding free energy calculations and support stable ligand–protein interactions within the active site.

## 4. Discussion

Matrix metalloproteinase-9 (MMP-9) plays a central role in extracellular matrix degradation and blood–brain barrier (BBB) disruption during cerebral ischemia, particularly in the early acute phase following ischemic insult [37]. However, clinical translation of MMP inhibitors has historically been limited by poor selectivity, suboptimal pharmacokinetics, and off-target toxicity [38]. The present study aimed to address this gap by applying an integrated computational workflow to identify structurally plausible MMP-9–binding compounds and to characterize their dynamic stability within the catalytic domain. Rather than proposing a disease-ready therapeutic, our objective was to prioritize chemically tractable scaffolds with favourable interaction profiles that may serve as starting points for further optimization.

Using a structure-based pharmacophore model derived from the crystallographic structure of MMP-9, we performed virtual screening, docking refinement, molecular dynamics simulations, and end-point binding free energy calculations. Importantly, the pharmacophore hypothesis was not constructed arbitrarily; interaction features were extracted from crystallographic ligand–protein interactions and validated using active–decoy benchmarking (ROC AUC = 0.70). It confirms performance above random selection and supports the utility of the model as a filtering step prior to docking.

Docking analysis demonstrated that several screened compounds occupied the catalytic cleft in orientations comparable to the co-crystal inhibitor. MMP-9 is a zinc-dependent metalloprotease, and effective inhibition typically involves interaction within proximity to the catalytic Zn² ⁺ ion coordinated by conserved histidine residues (His401, His405, His411) [38–40]. The identified hit compound, CHEMBL3990662, adopted a binding pose overlapping with the reference ligand and maintained interactions near the zinc-associated catalytic environment. Unlike classical broad-spectrum MMP inhibitors that rely on strong zinc-chelating hydroxamate groups often associated with poor selectivity and musculoskeletal side effects, the present compound appears to stabilize the catalytic pocket through a combination of hydrogen bonding and hydrophobic contacts. This binding mode may offer opportunities for achieving selective modulation rather than indiscriminate zinc chelation, though this hypothesis requires experimental validation.

Molecular dynamics simulations further supported stable accommodation of CHEMBL3990662 within the catalytic domain. Compared to the co-crystal ligand, the hit compound demonstrated comparable or slightly reduced backbone RMSD values over 200 ns, indicating structural stability without inducing major conformational disruption. RMSF and DCCM analyses revealed that fluctuations were primarily confined to solvent-exposed loop regions rather than catalytic residues, suggesting preservation of active-site architecture. PCA and free energy landscape (FEL) analyses indicated sampling of multiple low-energy conformational basins, consistent with dynamic but stable ligand engagement. While the variance distribution differed between systems, no evidence of destabilizing transitions or large-scale unfolding was observed.

End-point binding free energy calculations (MM/GBSA and MM/PBSA) showed slightly more favourable total binding energies for CHEMBL3990662 relative to the co-crystal ligand. The enhanced electrostatic contribution observed in the hit complex may reflect stronger stabilization within the catalytic cleft. Per-residue decomposition identified contributions from residues such as His117, Tyr139, Arg140, Val114, and Leu134, which are positioned near substrate-recognition regions. These interaction patterns are broadly consistent with reported structural studies of MMP-9 inhibitors that emphasize stabilization within the S1′ pocket and adjacent hydrophobic regions [39,41]. However, the energetic differences between complexes are moderate and should not be overinterpreted as definitive evidence of superior binding affinity.

From a translational perspective, it is important to contextualize MMP-9 inhibition in cerebral ischemia. MMP-9 upregulation occurs early after stroke and contributes to acute BBB disruption, whereas later phases may involve tissue remodelling processes [42,43]. Therefore, timing and reversibility of inhibition are critical considerations. The present computational study does not address pharmacodynamic timing, selectivity across MMP isoforms, or systemic safety. Furthermore, while BBB penetration was predicted computationally, high TPSA values in some compounds suggest that passive permeability may require optimization. Thus, although the identified scaffolds demonstrate favourable in silico binding characteristics, their relevance to stroke therapy remains hypothetical until validated experimentally in cellular and in vivo models.

All findings are based on computational modelling and depend on the accuracy of force fields, docking scoring functions, and continuum solvation approximations. MM/GBSA and MM/PBSA methods provide relative ranking rather than absolute binding free energies and selectivity against other MMP family members was not assessed, and broad-spectrum inhibition remains a concern in metalloproteinase drug discovery.

Future studies should include experimental enzyme inhibition assays to confirm activity, selectivity profiling across MMP isoforms, and evaluation of zinc-binding geometry using higher-level quantum mechanical calculations. Additionally, structure–activity relationship (SAR) optimization to modulate polarity, permeability, and metabolic stability would be necessary prior to disease-level consideration. In the context of cerebral ischemia, evaluation in BBB integrity models and time-dependent inhibition studies would be particularly informative.

In summary, this study provides a structured computational framework for prioritizing MMP-9–binding compounds and characterizing their structural and energetic stability within the catalytic domain. While the results support stable in silico binding of CHEMBL3990662, the findings should be interpreted as hypothesis-generating rather than conclusive evidence of therapeutic efficacy. Further experimental validation is required to determine whether these scaffolds can be developed into selective and pharmacologically viable MMP-9 inhibitors.

## 5. Conclusion

In this study, an integrated computational workflow combining pharmacophore modeling, virtual screening, molecular docking, molecular dynamics simulation, and MM/GBSA/MM/PBSA calculations was applied to prioritize potential MMP-9–binding compounds. Among the screened candidates, CHEMBL3990662 demonstrated favorable binding interactions within the catalytic pocket, stable accommodation during 200 ns molecular dynamics simulation, and comparable binding free energy estimates relative to the co-crystal ligand. These findings support the structural plausibility of the identified scaffold as a potential MMP-9–binding compound. However, the results are based solely on computational analyses, and experimental validation will be necessary to confirm inhibitory activity, selectivity, and therapeutic relevance. The present work provides a systematic in silico framework that may guide future optimization and experimental evaluation of MMP-9 inhibitors.
